# *Cordyceps militaris*—Fruiting Bodies, Mycelium, and Supplements: Valuable Component of Daily Diet

**DOI:** 10.3390/antiox11101861

**Published:** 2022-09-21

**Authors:** Karol Jędrejko, Katarzyna Kała, Katarzyna Sułkowska-Ziaja, Agata Krakowska, Piotr Zięba, Krystian Marzec, Agnieszka Szewczyk, Agnieszka Sękara, Jolanta Pytko-Polończyk, Bożena Muszyńska

**Affiliations:** 1 Department of Pharmaceutical Botany, Faculty of Pharmacy, Jagiellonian University Medical College, 9 Medyczna Street, 30-688 Kraków, Poland; 2 Department of Inorganic and Analytical Chemistry, Faculty of Pharmacy, Jagiellonian University Medical College, 9 Medyczna Street, 30-688 Kraków, Poland; 3 Department of Horticulture, Faculty of Biotechnology and Horticulture, University of Agriculture in Kraków, 29 Listopada 54, 31-425 Kraków, Poland; 4 Chair and Department of Integrated Dentistry, Faculty of Medicine, Jagiellonian University Medical College, 4 Montelupich Street, 31-155 Kraków, Poland

**Keywords:** cordycepin, antioxidant compounds, artificial digestive juices, edible mushrooms, mycelial cultures, food supplements, bioavailability

## Abstract

*Cordyceps militaris* has long been used in Eastern medicine for alleviating fatigue and as an immunostimulant. The present study aimed to determine the content of biologically active substances (bioelements and organic compounds), the total phenolic content, and the antioxidant activity of fruiting bodies (commercially available and self-cultivated), mycelia, and two food supplements. The results show that substrate composition and cultivation method had an influence on the properties of mushroom materials. An important aspect of the study is the estimation of the content of bioactive substances present after extraction into digestive juices in the artificial gastrointestinal tract model, which can allow for determining the amount of these substances that is potentially bioavailable for the human body. The best results for cordycepin (81.4 mg/100 g d.w.) and lovastatin (53.6 mg/100 g d.w.) were achieved for commercially available food supplements. Furthermore, after digestion in artificial intestinal juice, the highest amount of cordycepin was determined in the fruiting bodies from commercially obtained (25.9 mg/100 g d.w.) and self-cultivated mushroom (25.8 mg/100 g d.w.). In conclusion, the mycelium and fruiting bodies of *C. militaris* are ideal food supplements and pharmaceutical agents and can serve as a good source of prohealth substances potentially bioavailable for humans.

## 1. Introduction

*Cordyceps* spp. belonging to the Ascomycota group (family *Cordycipitaceae*) have long been used in Asian ethnomedicine as a natural agent for reducing tiredness and stimulating the immune system of the human body [1]. *Cordyceps militaris* (L.) Link (Scarlet Caterpillar Club) can be found on soil-dwelling larvae and pupae, which are the most common type of nocturnal butterflies, in light forests or on their edges. Studies on the fruiting bodies and mycelia of *C. militaris* have shown the presence of biologically active substances, such as γ-aminobutyric acid, ergothioneine, sterols (ergosterol), statins (lovastatin), phenolic compounds (including phenolic acids and flavonoids), vitamins, and bioelements [2,3]. Selenium is the bioelement in the fruiting bodies of *C. militaris*, where it occurs in an organic form or in combination with L-methionine (selenomethionine) or L-cysteine (selenocysteine) [4,5]. In addition to the aforementioned compounds, new bioactive components were isolated from fruiting bodies of *C. militaris*, such as cordyrrole A and cordyrrole B. The structure of cordyrrole A is based on a pyrrole ring with an N-1 substituent as 2-peperidone ring, as well as aldehydes and hydroxymethyl moieties at the C-2 and C-5 positions, respectively. In turn, cordyrrole B is a derivative of adenosine with an ester group. These substances are responsible for improving metabolic parameters [6]. Cordycepin (3′-deoxyadenosine), which is one of the most characteristic compounds of *C. militaris*, is a structural analog of the adenosine nucleoside. Cordycepin can occur in fruiting bodies as free-form or bound with the saccharide unit (glycoside), e.g., arabinoside [7,8]. Recent in vitro and in vivo tests on *C. militaris* have proven that cordycepin exhibits antitumor, immunostimulatory, anti-inflammatory, antiviral, and ergogenic activity [8]. In addition, *C. militaris* has been shown to induce apoptosis in ovarian cancer cells and its antitumor activity involves an increase in the levels of TNF-α, TNFR1, NF-κB, caspase-3, and caspase-9 and a reduction in Bcl-2 and Bcl-xL [9]. The antitumor activity of cordycepin and their derivatives such as NUC-7738, has also been confirmed in preclinical and clinical studies [10]. A study on a group of healthy adult males supplemented with 1.5 g of *C. militaris* daily for 4 weeks indicated enhanced immunostimulatory activity due to an increase in the levels of IL-2, IL-12, NK, TNF-α, and IFN-γ [11]. In growing pigs fed a diet containing 2 g *C. militaris* spent mushroom per 1 kg of feed, increases in immunoglobulin A and immunoglobulin G secretion have been shown and antioxidant capacity was supported, leading to increased glutathione peroxidase activity and decreased malondialdehyde (MDA) level in collected blood samples [12]. Research studies conducted in India revealed that cordycepin can be potentially used in the treatment of COVID-19, as this molecule exhibited strong chemical interactions with SARS-CoV-2 [13,14]. Additionally, in a study on an animal model, an improvement in exercise performance was observed in mice fed with *C. militaris* extract (cordycepin concentration 2.33 mg/g) for 12 weeks. The authors found that increased production of ATP correlated with the increased concentration of adenosine monophosphate-activated protein kinase (AMPK) and phosphocreatine [15]. During the 1993 sport event, the consumption of *Cordyceps* mushrooms gained a lot of attention as players consuming these mushrooms achieved good results in sports (especially in running) [16]. However, only a few human studies concerning supplementation with a mushroom mixture (containing, among others, *C. militaris*) have shown an improvement in tolerance to high-intensity exercises [17,18]. Additionally, in the latest research in a non-randomized controlled pilot trial, consumption of *C. militaris* fruiting bodies (as powder in the form of a capsule) by patients with benign prostatic hyperplasia (BPH) contributed to improve the urinary flow, alleviating micturition symptoms and decreasing size of the prostatic gland [19].

The aim of this study was to obtain in vitro cultures of *C. militaris* in aerated bioreactors with a carbon dioxide removal system and thus produce mushroom material with a potential biological effect. It also aimed to determine the content of biologically active substances in the mycelium of *C. militaris* and compare the estimated content with that in the fruiting bodies obtained from the cultivated biomass and from commercial origin, media, and two food supplements containing powdered mushroom material of the tested species. This study is the first to identify indole compounds in mycelium from in vitro cultures, fruiting bodies, medium obtained after cultivation, and selected commercial preparations containing *C. militaris*. Another important aspect of the study is the estimation of bioactive substances (bioelements and organic compounds) after extraction into digestive juices in the artificial digestive tract model, which can allow for determining the amount of these substances potentially bioavailable for the human body. Fruiting bodies and the mycelium of *C. militaris* obtained from optimized in vitro cultures can be used as functional food and as standardized material for the production of food supplements and potential drugs.

## 2. Materials and Methods

### 2.1. Reagents

The following reagents were used for the mineralization of lyophilized biomass, fruiting bodies, and media: 65% HNO_3_ Suprapur^®^ and 30% H_2_O_2_ Suprapur^®^ from Merck (Darmstadt, Germany). Bioelement content standards (Ca, Cu, Fe, K, Mg, Mn, Na, Zn) of 1 g/L concentration were purchased from the District Measurements Office (Łódź, Poland). Artificial digestive juices were prepared using the following: HCl and pancreatic extract from Merck (Darmstadt, Germany); bile salts and pepsin from BTL (Łódź, Poland); CaCl_2_ and MgCl_2_ from Chempur (Kraków, Poland); NaCl from Pol-Aura (Dywity, Poland); NaHCO_3_ from PPH Golpharm (Kraków, Poland); and Na_2_HPO_4_, KHCO_3_, K_2_HPO_4_, and citric acid from Avantor Performance Materials Poland (Gliwice, Poland). HPLC (high-performance liquid chromatography)-grade standards of cordycepin, lovastatin, ergothioneine, L-phenylalanine, sterols, and indole and phenolic compounds were obtained from Merck (Darmstadt, Germany). Analytical-grade methanol, acetic acid, phosphoric acid, and ammonium acetate were purchased from Chempur (Gliwice, Poland). HPLC-grade methanol, acetonitrile, and KH_2_PO_4_ were purchased from Merck (Darmstadt, Germany). Water (quadruple-distilled) with a conductivity of less than 1 µS/cm was obtained using an S2-97A2 distillation apparatus from Chemland (Stargard, Poland).

### 2.2. Digestive Juices Preparation

Artificial digestive juices, namely, saliva, gastric juice, and intestinal juice, were prepared as previously described [20,21,22].

Artificial saliva (pH 6.7) was prepared by mixing 100 mL of 24 mmol/L Na_2_HPO_4_, 100 mL of 1.5 mmol/L MgCl_2_, 6 mL of 25 mmol/L citric acid, 100 mL of 150 mmol/L KHCO_3_, 100 mL of 25 mmol/L KH_2_PO_4_, and 100 mL of 15 mmol/L CaCl_2_ in quadruple-distilled water to a volume of 1000 mL. This mixture is devoid of enzymes that are naturally present in human saliva [20].

Artificial gastric juice (pH 2) was prepared in accordance with the Polish Pharmacopoeia. First, 3.2 g of pure pepsin and 2.0 g of NaCl were dissolved in quadruple-distilled water. Then, 80 mL of 1 M HCl was added to achieve the appropriate pH, and the solution was made up to 1000 mL with quadruple-distilled water [22].

Artificial intestinal juice (pH 8) was prepared by mixing 0.02 g pancreatic extract, 0.125 g bile salts, and 8.4 g NaHCO_3_ in quadruple-distilled water to a volume of 1000 mL [21].

### 2.3. Mushroom Material

**Figure 1:**
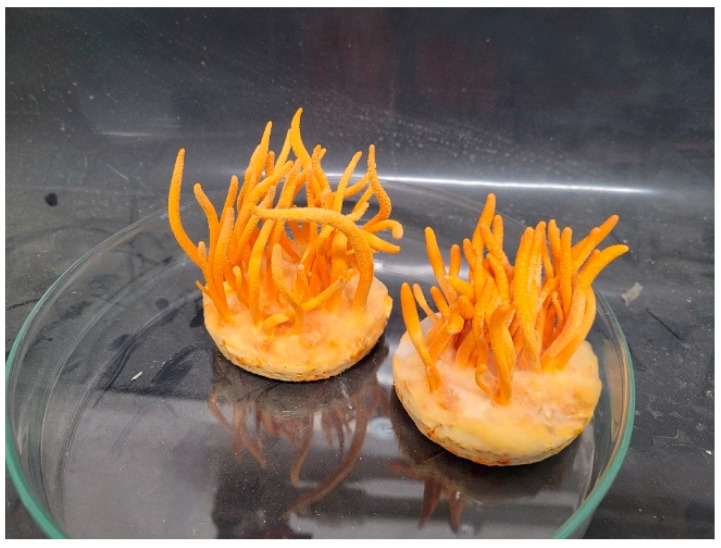
Self-cultivated *Cordyceps militaris* (photo by Piotr Zięba).

Purchased and self-cultivated (mycelium from in vitro cultures and fruiting bodies) materials of *C. militaris* were used in the study (Figure 1).

Mycelium, self-cultivated fruiting bodies and fruiting bodies from commercial crops were obtained in 2021. To compare the quality of commercially available and self-cultivated mushroom material, dried fruiting bodies obtained from commercial crops (ChinaLover Store, Shenzhen, China) and two food supplements: food supplement 1 (FS1) (Mountain Rose Herbs, Eugene, OR, USA; powdered mycelia) and food supplement 2 (FS2) (Noomadic Herbals, Markham, ON, Canada; extract standardized for 30% β-d-glucans content) were used. The food supplements had an expiry date of 2024. Fruiting bodies and mycelium from in vitro cultures were also obtained from own cultivation. Substrate composition was analyzed both before and after cultivation of the fruiting bodies. All the samples of *C. militaris* were deposited in the Department of Pharmaceutical Botany, Faculty of Pharmacy, Jagiellonian University Medical College.

### 2.4. Mycelium from In Vitro Cultures

The mycelia of *C. militaris* were cultured on a solid medium according to Oddoux with some modifications (with appropriate concentrations of micro- and macroelements) [23]. The glass, media, and instruments used for cultivation were sterilized in an ASVE-type vertical steam sterilizer (Asve, Warsaw, Poland) at 0.1 MPa and 121 °C for 20 min. The in vitro culture was passaged under sterile conditions in a laminar chamber (Polonium, type K-21, Poznań, Poland) at an airflow of 0.45 m/s. The biomass of in vitro cultures on the solid medium was passaged by adding 0.1 g of inoculum to 250 mL of liquid medium in 500 mL Erlenmeyer flasks. The in vitro cultures were kept for 14 days on a TOS-6048FD laboratory shaker with orbital motion (EnviSense, Lublin, Poland). To achieve efficient growth of mushroom biomass, the mycelium from cultures in Erlenmeyer flasks was transferred to a bioreactor containing 10 L Oddoux medium, and the culture was mixed by air inflow via sterile antibacterial filters. The carbon dioxide formed during mycelial growth was removed. The cultures were maintained at 25 ± 2 °C under 500 lx light for 10 h and in dark for 14 h. After 10 days of growth in the bioreactor, the mycelia from in vitro cultures were separated by filtration using a Pyrex Buchner funnel with Grade 1 Whatman^®^ qualitative filter paper (Merck, Darmstadt, Germany). The mycelia were washed several times with quadruple-distilled water, frozen, and lyophilized at −40 °C (Freezone 4.5 lyophilizer, Labconco, Kansas City, MO, USA). After lyophilization, the mycelia were powdered in an agate mortar. The growth of mycelia was evaluated as final biomass after cultivation in uniform volumes of liquid medium.

### 2.5. Fruiting Bodies from Self-Cultivation

The growth medium used in this experiment was brown rice, which is the most widely used substrate for the cultivation of *C. militaris*. For cultivation, a total of 10 polypropylene containers (500 mL volume) were filled with 20 g of brown rice and 32 mL of medium (consisting of 40 g/L glucose, 5 g/L peptone, 1.5 g/L MgSO_4_ × 7H_2_O, and 1.5 g/L K_2_HPO_4_) optimized to achieve effective biomass growth [24]. The containers were then closed, and holes were made in the upper part through which filters were glued to ensure air exchange within. Then, the containers were autoclaved at 121 °C to avoid biological contamination. Finally, the containers were inoculated under a laminar chamber with previously prepared mycelium from in vitro cultures. The prepared containers were placed at a temperature of 20 °C for 2 weeks in dark, until the entire medium was overgrown with white mycelium. After mycelial growth, the containers were exposed to light at an intensity of 500 lx, while the temperature was maintained throughout at 20 °C. After 2 weeks, the first fruiting bodies appeared. Harvesting was performed after 6 weeks of inoculation, when mature ascus with spores appeared on the fruiting bodies.

### 2.6. Analysis of Bioelements

The *C. militaris* materials were wet-mineralized in the closed system in Magnum II (Ertec, Wrocław, Poland), with 6 mL of concentrated 65% HNO_3_(V) and 2 mL of 30% H_2_O_2_ solution. Then, the whole content was transferred to Teflon vessels and mineralized as described by Krakowska et al. (2020) [25]. The concentrations of selected bioelements in the mineralized materials were determined by flame atomic absorption spectrometry (FAAS) using an iCE 3500 spectrometer (Thermo Scientific, Waltham, MA, USA). The determination of bioelements was carried out in accordance with the Polish certified reference material for multielemental trace analysis (mixed polish herbs, INCT-MPH-2; Institute of Nuclear Chemistry and Technology, Warszawa, Poland). Each of the prepared analytical samples was tested in three independent repetitions, and the results were presented as mean values with standard deviation.

### 2.7. Preparation of Extracts for Organic Compounds Determination

To obtain biomass samples (each weighing 4 g) for the determination of organic compounds, the fruiting bodies, medium obtained after self-cultivation, mycelium from in vitro cultures, and food supplements of *C. militaris* were lyophilized and homogenized in an agate mortar. The material was then mixed with methanol and extracted three times for 30 min in an ultrasonic bath at 49 kHz (Sonic-2, Polsonic, Warszawa, Poland). The final volume of the extracted solutions was 300 mL. The extracts were evaporated in crystallizers, and the remaining residue was dissolved in 10 mL HPLC-grade methanol and filtered through membrane filters (Millipore Millex^®^ GP 0.22 µm; Merck, Darmstadt, Germany). The prepared extracts were used for further analyses.

### 2.8. Mycochemical Analysis

#### 2.8.1. Indole Compounds, Lovastatin, Ergothioneine, Phenolic Compounds, L-Phenylalanine, Sterols

Analyses of indole compounds, lovastatin, and ergothioneine were carried out using a Hitachi HPLC system (Merck, Tokyo, Japan). The content of these compounds was determined by reverse-phase HPLC (RP-HPLC) using a UV detector operated at λ = 280 nm for indole compounds, 238 nm for lovastatin and 257 nm for ergothioneine. The analyses of indole compounds, lovastatin and ergothioneine, were performed as previously described by Muszyńska et al. (2017), Kała et al. (2020), and Krakowska et al. (2020), respectively [25,26,27].

Analyses of phenolic compounds, L-phenylalanine, and sterols were carried out using a Hitachi-Merck HPLC VWR liquid chromatograph (Darmstadt, Germany). All analyses were performed by RP-HPLC using a diode array detector (DAD) operated at the UV range of 200–400 nm. The content of phenolic compounds and L-phenylalanine was determined as previously described by Kała et al. (2021), and the content of sterols as described by Sułkowska-Ziaja et al. (2018) [28,29].

The content of all analyzed substances was expressed in mg/100 g dry weight (d.w.).

#### 2.8.2. Cordycepin

The content of cordycepin was analyzed by RP-HPLC as described by Kaushik et al. (2020) with some modifications [30]. The analysis was performed using a Hitachi HPLC system (Merck, Tokyo, Japan) equipped with a type L-7100 pump. The Purospher^®^ RP-18 column (4 mm × 200 mm, 5 µm) was heated at 25 °C, and the UV detector was operated at λ = 261 nm. The mobile phase consisted of a mixture of methanol and ultrapure water (10:90) with 10 mmol/L KH_2_PO_4_ dissolved in it and the flow rate was set at 1.0 mL/min. The quantitative analysis of cordycepin was performed based on a calibration curve (1, 0.5, 0.25, 0.125, and 0.0625 mg/mL) assuming the linear correlation between the area under the peak and the concentration of the reference standard (the same as for the previously described organic compounds). The content of cordycepin was expressed in mg/100 g d.w.

#### 2.8.3. Determination of Antioxidant Activity Using a DPPH Assay

The antioxidant activity of the analyzed mushroom material was determined using the DPPH (1,1-diphenyl-2-picrylhydrazyl) radical (Sigma Aldrich, St. Louis, MO, USA). Briefly, 0.1 g of powdered samples of the fruiting bodies, media, in vitro cultures, and food supplements were weighed in three repetitions and mixed with 4.9 mL of 0.1 mM DPPH solution in 80% methanol. The prepared solution was incubated in darkness at room temperature for 30 min. After incubation, the solution was analyzed as previously described by Krakowska et al. (2020) with a Helios Beta UV-VIS spectrophotometer—λ = 517 nm (Thermo Fisher Scientific Inc., Waltham, MA, USA) [25]. The DPPH reduction was calculated using the formula, AA (%) = ((A0 − A1)/A0) × 100, where AA is the antioxidant activity (%), A0 is the absorbance of the blank sample, and A1 is the absorbance of the examined concentration.

#### 2.8.4. Total Phenolic Content

The total phenolic content was determined by the Folin–Ciocalteu method as described by Djeridane et al. (2006) [31]. Briefly, 0.1 g of the mushroom material was weighed and placed in a mortar and triturated in 10 mL of 80% methanol. The obtained extract was poured into plastic tubes and centrifuged for 10 min (6000 rpm). After decanting, 0.1 mL of sample was added to 0.5 mL (1/10 dilution) of the Folin–Ciocalteu reagent and 1 mL of distilled water. After 2 min 1.5 mL solution of 20% Na_2_CO_3_ was added. Solution was shaken and incubated for 2 h in the darkness at room temperature. The absorbance of the extract was measured at 750 nm using the UV-VIS Helios Beta spectrophotometer (Thermo Fisher Scientific Inc., Waltham, MA, USA). The total phenolic content in the fruiting bodies, media, in vitro cultures, and food supplements was calculated based on the calibration curve of gallic acid and expressed as gallic acid equivalents (GAE) in mg/g of the sample.

### 2.9. Extraction in Digestive Juices

Fruiting bodies, mycelial cultures, and food supplements were analyzed to determine their potential bioavailability for humans. For the analysis, 0.5 g of the mushroom material was weighed, ground in an agate mortar, and moistened with a solution of artificial saliva to predigest the material. Then, the material was digested in gastric juice at 37 °C for 60 min to mimic the natural digestion of food in the stomach. Gastric juice digestion was carried out in the Gastroel-2014 apparatus constructed specifically to imitate the standard conditions prevailing in the human gastrointestinal tract [32]. After 60 min, the solution was decanted and the intestinal juice solution was added to the remaining biomass. The material was incubated in the intestinal juice under the same conditions for 150 min. The decanted solutions were then filtered using membrane filters (Millipore Millex^®^ GP 0.22 µm; Merck, Darmstadt, Germany). One half of the obtained solutions were used for estimating the content of bioelements, and the other half, after evaporation and dissolution in HPLC-grade methanol, was transferred to Eppendorf tubes using sterile syringes and syringe filters (0.22 µm) to determine the content of organic compounds.

### 2.10. Statistical Analysis

Each of the tested samples was analyzed in three independent repetitions. The results of the analyses were presented as mean values with standard deviation (SD). Statistical significance was set at *p* < 0.05. The composition of bioelements and organic compounds was evaluated using Tukey’s test (ANOVA analysis of variance). Chemometric analysis was conducted in Statgraphics Centurion XVIII software. The correlations between the studied variables in the analyzed data set were determined using chemometric tools such as Cluster Analysis (CA) and Principal Component Analysis (PCA).

## 3. Results and Discussion

The material analyzed in this study was chosen due to its diversity. Self-cultivated fruiting bodies, media, and mycelial cultures, as well as commercially available fruiting bodies and food supplements, were selected to compare their qualitative and quantitative composition, potential bioavailability, and therapeutic properties. The yield of mycelium from the in vitro cultures grown on the Oddoux medium in aerated bioreactors was on average 16–17 g d.w./L. The dynamics of mycelial growth was twice as high as that observed in nonaerated liquid in vitro cultures [28,33].

### 3.1. Bioelements and Organic Compounds Analysis in Mushroom Material

The first stage of the study involved the analysis of the content of bioelements and organic compounds in the mushroom material. In the case of bioelements, the optimized conditions applied for the homogenization and mineralization of the obtained material, together with the use of the AAS method, allowed for a quick and effective analysis. The results obtained for the media before and after the cultivation indicated that, compared to the initial medium, the amounts of bioelements were much lower in the substrate after cultivation (Table 1).

This proves that the fruiting bodies of edible mushrooms efficiently absorb bioelements from the substrate and accumulate them in the mycelial structures. The bioelements Cu and Mn, which were found in small amounts before cultivation, were no longer present in the substrate after cultivation. Macronutrients such as Na and K, as well as Ca and Mg, were found in the highest amounts both in the initial medium and in the medium after cultivation. Among the micronutrients, Zn was dominant (Table 1).

Among the analyzed mushroom materials—fruiting bodies, mycelium from in vitro cultures, and food supplements—FS2 was the best source of Ca, Cu, and Mg (500, 2.95, and 817 mg/100 g d.w., respectively). In turn, the richest source of Fe was mycelial cultures (9.36 mg/100 g d.w.), and that of K, Mn, Na, and Zn (2025, 2.89, 1886, and 16.2 mg/100 g d.w., respectively) was commercially available fruiting bodies (Table 1). The amounts of Mg determined in both mycelial cultures (341 mg/100 g d.w.) and fruiting bodies (423 mg/100 g d.w.) were similar to those reported in previous studies, while the amounts of Zn, Ca, and Fe determined in this study significantly exceeded the previously determined amounts [2]. The available literature, however, presents differences in the determined contents of bioelements, which may be directly related to the variations in the composition of the substrate on which the fruiting bodies grew. In the study by Cohen et al. (2014), the amount of Mg determined was only 110 mg/100 g d.w., which is four times lower compared to that determined in the present study in self-cultivated and commercially available fruiting bodies [3]. Earlier analyses of bioelements in *C. militaris* showed that fruiting bodies and mycelia from the in vitro cultures of this species may also contain some amounts of S, Se, Li, or P [2,3,34].

The analysis of the content of organic compounds revealed that the mushroom material can be a good source of indole and phenolic compounds, sterols, as well as cordycepin, lovastatin, ergothioneine, or L-phenylalanine (Table 2).

The content of organic compounds in the medium after cultivation was also analyzed in order to verify whether such natural material can serve as a source of bioactive compounds and can be reused as a substrate of growth media. Recent studies have highlighted the reuse of mushroom substrates, particularly for the production of horticultural plants, vegetables, and fruits [35,36]. The medium overgrown with mycelium has been shown to be rich in 5-hydroxy-L-tryptophan, serotonin, and ergosterol. Pintathong et al. (2021) also determined the content of tryptophan in solid-based residues remaining after the harvest of fruiting bodies of *C. militaris* [37]. Certain amounts of cordycepin, lovastatin, and ergothioneine were also found in these residues, but only lovastatin was present in significantly lower amounts compared to other mushroom materials (1.90 mg/100 g d.w.). The content of cordycepin in the fruiting bodies of self-cultivated mushrooms was comparable to that determined in the medium (Table 2). The antioxidant activity of the medium was also better compared to that of mycelia from in vitro cultures, and even the tested food supplements. The analysis of the content of bioelements and organic compounds in the substrate after cultivation shows that the spent substrate can still serve as a valuable source of many bioactive substances (Table 1 and Table 2).

The mushroom material contained a significant amount of indole compounds with antidepressant and nootropic activity [38]. The highest content of 5-hydroxy-L-tryptophan (296 mg/100 g d.w.) was determined in FS1 and in the mycelium from in vitro cultures (206 mg/100 g d.w.). The amounts of 5-hydroxy-L-tryptophan determined in the fruiting bodies were comparable to those previously determined in six species of Pleurotus (67.5–194 mg/100 g d.w.) (Table 2) [25]. Similarly, the content of ergosterol was found to be high, especially in mycelial cultures (477 mg/100 g d.w.). Moreover, significant differences in the content of this compound were observed in the tested food supplements (155 mg/100 g d.w. in FS1 and 4.28 mg/100 g d.w. in FS2). Yang et al. (2009) determined a lower amount of ergosterol than that determined in the present study (9.37–30.7 mg/100 g d.w.) in commercially available *C. militaris* [39]. The content of α-tocopherol was the highest in FS1 (242 mg/100 g d.w.) and the lowest in FS2 (5.87 mg/100 g d.w.), which is analogous to ergosterol (Table 2). Among phenolic compounds, p-hydroxybenzoic acid was determined only in self-cultivated fruiting bodies and its amounts were similar to those previously determined [40]. Quercitrin and rutoside were found in self-cultivated fruiting bodies and the mycelia from in vitro cultures at an amount of 0.066–2.54 mg/100 g d.w. (Table 2). Ergothioneine was present at the highest amount in mycelial cultures (10.4 mg/100 g d.w.), as well as in self-cultivated or commercially available fruiting bodies. However, these amounts were smaller than that previously described (41.0 mg/100 g d.w.) [3]. In the case of lovastatin, higher content was determined in the tested food supplements, as well as in commercially available fruiting bodies [3]. The antioxidant activity was also the highest in fruiting bodies from commercial and own cultivation (Table 2). It should be noted that in previous studies, the antioxidant activity was investigated mainly based on polysaccharides from *C. militaris* [34,41].

One of the most frequently described compounds in *C. militaris* is cordycepin with proven antitumor, immunostimulating activity, but also supporting the efficiency of the human body [34]. In this study, the largest amounts of this compound were determined in the examined food supplements (60.4 mg/100 g d.w. in FS1 and 81.4 mg/100 g d.w. in FS2), as well as in fruiting bodies from commercial cultivation (57.5 mg/100 g d.w.). Other researchers have reported that the amount of cordycepin in fruiting bodies is in the range of 110–837 mg/100 g d.w. and in mycelial cultures about 175 mg/100 g d.w. [2,3]. Kang and Chamyuang et al. investigated the possibility of obtaining strains that can produce high amounts of cordycepin, as well as optimizing the cultivation or extraction conditions [42,43]. Depending on the medium used, the authors obtained on average 108–417 mg/100 g d.w. of cordycepin in fruiting bodies. In the strain with the highest content of cordycepin, the amount was determined at 663 mg/100 g d.w. [43]. Other researchers achieved an amount of 60–7740 mg/100 g d.w. of cordycepin in fruiting bodies (depending on the strain and substrate used), which is comparable with that determined in the present study (Table 2) [44].

### 3.2. Bioelements and Organic Compounds Analysis in Artificial Digestive Juices

To determine the actual amounts of bioelements and organic compounds available to the human body, a specially designed apparatus known as Gastroel-2014 was used to analyze the release of the tested material (mycelia from in vitro cultures, fruiting bodies, and commercially available food supplements) into artificial gastric and intestinal juices. The bioavailability of substances contained in the biomass obtained from the mycelial cultures was examined by determining the amount of substances extracted to digestive juices (artificial gastric juice in 60 min and artificial intestinal juice in 150 min). The content of analyzed bioelements determined in the initial material was found to be significantly higher than that potentially bioavailable, i.e., determined after in vitro-simulated gastrointestinal digestion (Table 1). Compared to intestinal juice, a higher amount of bioelements was determined in gastric juice, as shown by the previous studies [28,33]. After extraction into artificial gastric juice, the highest amount of valuable bioelements such as Cu, Fe, or Zn was obtained from mycelium from in vitro cultures. Fruiting bodies from commercial origin were the richest source of bioavailable Mn, Na, and K, while the highest amounts of bioavailable Ca and Mg were extracted from food supplements (FS1 and FS2, respectively) (Table 1). In the case of intestinal juice, the highest amounts of Ca and Mg were extracted from commercially available FS1 and FS2, and K and Mn from commercially available fruiting bodies. Furthermore, the largest amounts of Cu and Fe were released into intestinal juice from FS1, while self-cultivated fruiting bodies were found to be a rich source of Zn and Na (Table 1). These results indicate the need for more studies on the bioavailability of bioelements and organic compounds of *C. militaris* [45].

Similar to bioelements, most of the organic compounds were also extracted into artificial digestive juices. The analysis of L-tryptophan showed that a higher content of this compound was extracted in digestive juices compared to that in methanol extracts, which may indicate its potential bioavailability from mushroom material. Importantly, 5-hydroxy-L-tryptophan was extracted from all the analyzed samples at an amount of 10.4–37.3 mg/100 g d.w. in gastric juice and 3.40–22.3 mg/100 g d.w. in intestinal juice. FS1 was identified to be the best source of bioavailable 5-hydroxy-L-tryptophan (Table 2). Among the analyzed indole compounds, trace amounts of melatonin were also extracted into digestive juices. A similar tendency was observed in the release of ergosterol peroxide and lovastatin. Due to its chemical structure, only small amounts of ergosterol (4.49–8.14 mg/100 g d.w.) were released into digestive juices. In the case of a strong antioxidant, i.e., ergothioneine, a larger amount was extracted into artificial gastric juice (2.82–28.3 mg/100 g d.w.) and smaller amount into artificial intestinal juice (1.43–8.53 mg/100 g d.w.). A small amount of cordycepin was determined in the methanol extracts of mycelium from in vitro cultures, but after extraction in artificial gastric juice some amounts of it were extracted only from this material. In the intestinal juice, cordycepin was determined at an amount of 7.03–25.9 mg/100 g d.w., and its richest source was fruiting bodies (both self- and commercially cultivated) (Table 2). Nevertheless, it has been shown that the bioavailability of cordycepin from mushroom material is low [45]. In the case of L-phenylalanine, the largest amounts were extracted from mycelium from in vitro cultures, and the methanolic extracts of this material were also found to be the best source (Table 2). Scientific studies have proven that, in addition to the high content of bioactive compounds in the in vitro digestion model, *C. militaris* may have a positive effect on modulating the composition of the intestinal microbiota [46].

### 3.3. Chemometric Analysis

The content of macro- (Ca, K, Mg, Na) and microelements (Cu, Fe, Mn, Zn) and organic compounds (including L-tryptophan, 5-hydroxy-L-tryptophan, melatonin, L-phenylalanine, ergosterol, ergosterol peroxide, ergothioneine, cordycepin, lovastatin, quercitrin, rutoside) was determined in the present study. This allowed for creating an extensive data set, which included both the results of the analysis of the mushroom material and the results of the analysis of their extracts obtained in artificial digestive juices. Interpretation of data in a large data set is complex. Therefore, to understand the correlation between the type of the tested material (mycelium from in vitro cultures, fruiting bodies from commercial cultivation, self-cultivated fruiting bodies, FS1, FS2), a chemometric analysis of the content of bioelements was carried out.

The objects considered in the analysis were samples of the *C. militaris* material, which were described using specific parameters—bioelements marked in them. The data variability in the analyzed set of results indicated that the use of chemometric analysis to obtain detailed information on the relationships between the tested samples was fully justified. Two commonly known methods of chemometric analysis—CA and PCA—were used in the present study.

**Figure 2:**
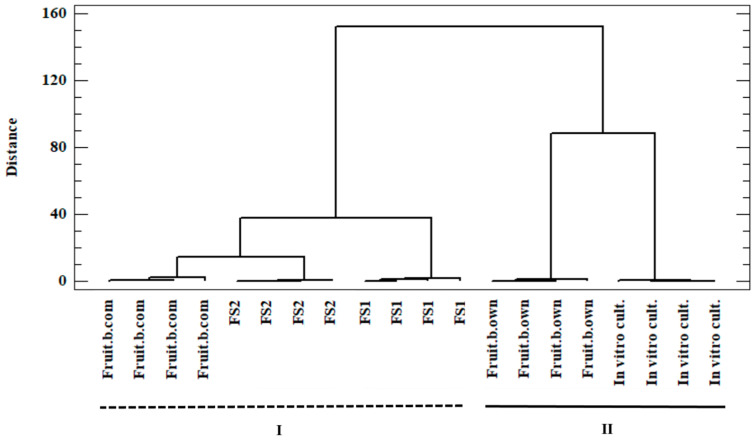
Dendrogram showing the existing correlation in the investigated data set between the analyzed objects—samples from *C. militaris*: In vitro cult.—mycelium from in vitro cultures, Fruit.b.own—fruiting bodies from own cultivation, Fruit.b.com—fruiting bodies from commercial cultivation, FS1—food supplement 1, FS2—food supplement 2 (Squared Euclidean, Ward’s algorithm).

The use of CA allowed for determining the correlations between the considered objects, i.e., samples of the *C. militaris* material. In this method, the correlation between the studied objects is evidenced by their mutual location in the multidimensional space under consideration. On the other hand, the dendrogram (Figure 2), commonly known as a tree, is a graphic interpretation of the CA method used.

The numerical axes presented on this graph do not correspond to the axes in the Cartesian system (x axis corresponds to the considered objects—*C. militaris* samples, and y axis corresponds to the distances between the objects calculated using the Ward agglomeration method and the objects with significant similarity, i.e., clusters).

Based on CA (Figure 2), two main clusters (cluster I and II) were distinguished in the analyzed data set. Cluster I includes the samples of *C. militaris* from commercial cultivation and food supplements prepared from this species, while cluster II includes the samples of *C. militaris* from own cultivation and mycelium from in vitro cultures. The presence of a given object in a given cluster proves a high correlation between the considered objects classified under it (as confirmed by the short arms in the presented dendrogram; Figure 2). In this case, this similarity is related to the composition of the sample, i.e., the content of the bioelements tested in it. The conducted analysis revealed the differences between the tested samples of the material, despite the fact that they came from one species (*C. militaris*), as evidenced by their inclusion in separate clusters. These differences may be attributed to the different physicochemical conditions of cultivation as well as by the method used for biomass preparation, and suggest that there may be discrepancies in the determined concentration of bioelements in the analyzed sample.

The extensive results from the determination of the concentrations of bioelements and organic compounds constituting the input data set made their interpretation in the multidimensional space complicated. Therefore, a second analytical method, PCA, was used in the study, which reduced the data set to the necessary minimum such that their mutual correlation and visualization could be defined. The output data were thus replaced with the so-called main components (a linear combination of input variables and the loads assigned to them, which correspond to the correlation size in the set). The analysis showed that three components, PC1, PC2, and PC3 (data set described by the bioelements content in *C. militaris* samples), explained 96.84% of changes in the examined data set. On the other hand, 95.47% of changes were described by the three main components PC1, PC2, and PC3 (a set of data defined by the content of organic compounds determined in the *C. militaris* samples).

**Figure 3:**
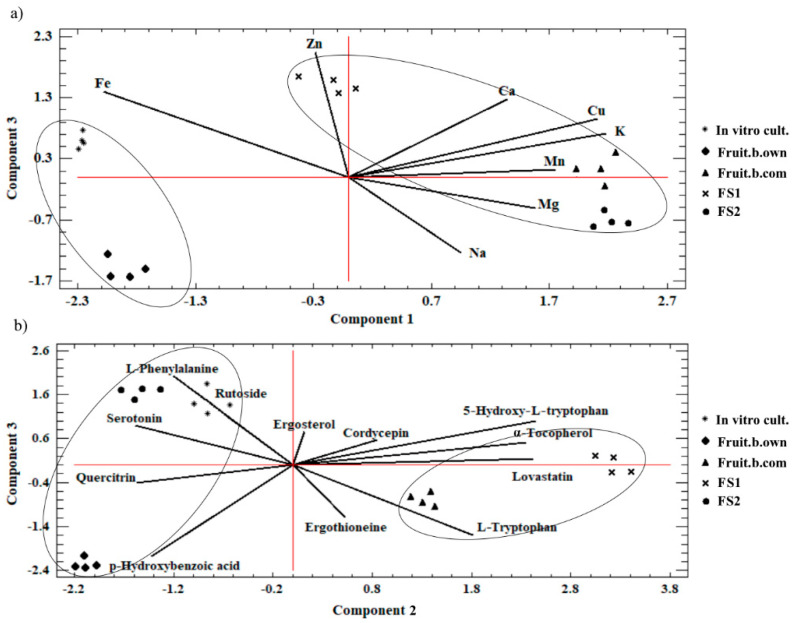
Graph showing the correlation between the analyzed *C. militaris* samples in relation to (**a**) the content of bioelements and (**b**) the content of organic compounds determined in them.

The analysis of the graphs presented in Figure 3 revealed the existence of two clusters.

The first included the samples of *C. militaris* from own cultivation and mycelium from in vitro cultures, and the second included samples from commercial cultivation and food supplements containing *C. militaris*. The presence of an object in a given cluster confirms the significant similarity of objects within the analyzed cluster. The similarity between the given objects (mycelium from in vitro cultures and self-cultivated *C. militaris*) may have resulted from the fact that the mycelial cultures of *C. militaris* were used for self-cultivation of this mushroom species. In turn, the direction defined by the arms of the plot (Figure 3a,b) indicated the objects with the highest concentrations of bioelements (Figure 3a) or the analyzed organic compounds (Figure 3b). Considering the content of bioelements, it was found that the samples from in vitro cultures had the highest content of Fe, while FS2 and the fruiting bodies from commercial origin contained the highest amount of bioelements such as Mg, Na, or K. On the other hand, *C. militaris* samples from in vitro cultures and self-cultivated fruiting bodies had the highest content of organic compounds such as ergosterol, L-phenylalanine, rutoside, and quercitrin (Figure 3b). The fruiting bodies from commercially cultivated *C. militaris* as well as FS1 (second cluster; Figure 3b) had the highest content of α-tocopherol, lovastatin, or L-tryptophan.

**Figure 4:**
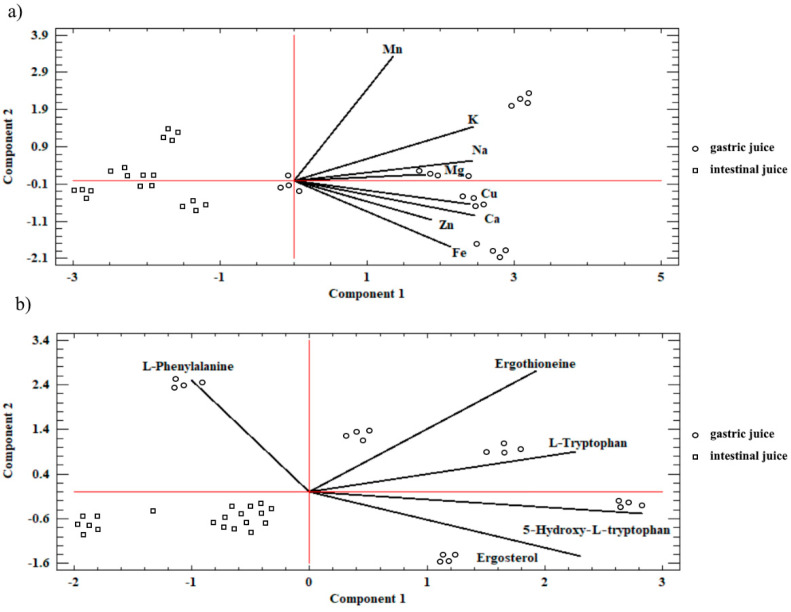
Plot of the similarity of *C. militaris* samples in relation to the place of their extraction in artificial digestive juices: (**a**) bioelements; (**b**) organic compounds.

Considering the analysis of the objects (samples from *C. militaris*), two clusters were distinguished based on the amount of extracted macro- and microelements and organic compounds in relation to the location of their extraction in the digestive system. As in other experimental studies which analyzed the samples of *Agaricus bisporus* or *Pleurotus* spp. mushrooms, it was found in the present study that the extraction of bioelements and organic compounds in the digestive system took place in a targeted manner (Figure 4a,b) [25].

The direction of the arms shown in Figure 4 indicates the place (artificial gastric juice) where the analyzed substances were the most extensively extracted. The extraction of the analyzed *C. militaris* samples in the artificial gastric juice may suggest less effective absorption of bioelements and organic compounds from these materials into the human organism as the absorption takes place mainly in the intestines in humans.

## 4. Conclusions

The analysis of bioelements and organic compounds in the present study shows, for the first time, not only the content but also the bioavailability of substances that are important for human health. Each of the analyzed materials—both from own cultivation and commercial cultivation—contained a slightly different amount of substances, but all the materials were found to be a valuable source of cordycepin, ergothioneine, as well as indole compounds, lovastatin, and bioelements. The contained active ingredients have shown numerous biological activities in many studies, such as antioxidant, antitumor, immunostimulant, ergogenic, and anti-inflammatory activity. In the case of cordycepin, the best potentially bioavailable source was both self-cultivated and commercially available fruiting bodies. In conclusion, it should be emphasized that not only food supplements but also mycelium from in vitro cultures and fruiting bodies of *C. militaris* can be a potential dietary and therapeutic material and a source of supplementation for the human body. Due to the possibility of optimizing the cultivation of fruiting bodies and obtaining mycelium with increased content of health-promoting substances, further research should be performed to obtain material for the production of therapeutics in the future.

## Figures and Tables

**Table 1:** Content of bioelements in mushroom material [mg/100 g d.w].

	Bioelements in Mushroom Material							
	Ca	Cu	Fe	K	Mg	Mn	Na	Zn
Mycelium from in vitro cultures	351 ± 5 ^b^	1.77 ± 0.06 ^d^	9.36 ± 0.27 ^b^	1371 ± 21 ^e^	332 ± 10 ^a^	nd	932 ± 15 ^f^	14.0 ± 0.8 ^c^
Fruiting bodies (own cultivation)	312 ± 8 ^c^	0.982 ± 0.071 ^f^	7.54 ± 0.51 ^c^	1306 ± 33 ^e^	398 ± 7 ^b^	0.687 ± 0.160 ^c^	1224 ± 25 ^c^	10.8 ± 0.6 ^b^
Medium before cultivation	504 ± 16 ^a^	1.41 ± 0.15 ^e^	12.2 ± 0.7 ^a^	1868 ± 23 ^b^	840 ± 5 ^d^	1.16 ± 0.09 ^b^	1568 ± 23 ^b^	20.4 ± 1.2 ^d^
Medium after cultivation	208 ± 12 ^d^	nd	2.06 ± 0.09 ^e^	231 ± 12 ^f^	413 ± 8 ^b^	nd	431 ± 12 ^g^	5.54 ± 0.79 ^a^
Fruiting bodies (commercial cultivation)	366 ± 9 ^b^	2.58 ± 0.19 ^b^	4.58 ± 0.07 ^d^	2025 ± 39 ^a^	412 ± 8 ^b^	2.89 ± 0.18 ^a^	1886 ± 32 ^a^	16.2 ± 0.9 ^c^
Food supplement 1	492 ± 13 ^a^	2.22 ± 0.15 ^c^	8.86 ± 0.73 ^b^	1630 ± 11 ^d^	523 ± 10 ^c^	1.06 ± 0.09 ^b^	996 ± 18 ^e^	14.6 ± 1.0 ^c^
Food supplement 2	500 ± 9 ^a^	2.95 ± 0.16 ^a^	4.10 ± 0.16 ^d^	1727 ± 33 ^c^	817 ± 11 ^d^	0.945 ± 0.046 ^bc^	1115 ± 17 ^d^	7.50 ± 0.56 ^a^
After Extraction in Digestive Juices								
Gastric Juice								
	Ca	Cu	Fe	K	Mg	Mn	Na	Zn
Mycelium from in vitro cultures	174 ± 8 ^b^	1.28 ± 0.11 ^a^	4.87 ± 0.47 ^a^	664 ± 17 ^c^	191 ± 9 ^cd^	nd	616 ± 7 ^b^	6.82 ± 0.75 ^a^
Fruiting bodies (own cultivation)	103 ± 4 ^d^	0.323 ± 0.014 ^c^	2.34 ± 0.27 ^c^	601 ± 25 ^c^	185 ± 12 ^d^	0.212 ± 0.011 ^c^	468 ± 29 ^c^	4.42 ± 0.21 ^bc^
Fruiting bodies (commercial cultivation)	141 ± 6 ^c^	0.912 ± 0.054 ^b^	3.50 ± 0.23 ^b^	1108 ± 47 ^a^	239 ± 6 ^b^	1.85 ± 0.15 ^a^	710 ± 31 ^a^	5.04 ± 0.20 ^b^
Food supplement 1	235 ± 10 ^a^	0.829 ± 0.051 ^b^	4.44 ± 0.47 ^a^	1006 ± 27 ^ab^	215 ± 10 ^bc^	0.567 ± 0.078 ^b^	382 ± 8 ^d^	5.13 ± 0.82 ^b^
Food supplement 2	160 ± 8 ^bc^	1.22 ± 0.04 ^a^	2.09 ± 0.16 ^c^	920 ± 63 ^b^	489 ± 11 ^a^	0.240 ± 0.011 ^c^	480 ± 33 ^c^	3.21 ± 0.55 ^c^
Intestinal Juice								
	Ca	Cu	Fe	K	Mg	Mn	Na	Zn
Mycelium from in vitro cultures	39.1 ± 1.7 ^c^	nd	1.46 ± 0.18 ^b^	210 ± 9 ^e^	39.6 ± 0.9 ^c^	nd	176 ± 6 ^d^	3.42 ± 0.15 ^ab^
Fruiting bodies (own cultivation)	49.7 ± 5.9 ^c^	nd	0.648 ± 0.168 ^c^	438 ± 14 ^b^	50.1 ± 4.6 ^c^	nd	301 ± 14 ^a^	3.99 ± 0.75 ^a^
Fruiting bodies (commercial cultivation)	67.4 ± 9.2 ^b^	0.379 ± 0.024 ^a^	0.455 ± 0.008 ^c^	583 ± 27 ^a^	33.3 ± 1.8 ^c^	0.752 ± 0.055 ^b^	250 ± 16 ^bc^	3.17 ± 0.27 ^ab^
Food supplement 1	97.5 ± 6.3 ^a^	0.524 ± 0.113 ^a^	3.23 ± 0.15 ^a^	336 ± 10 ^c^	85.0 ± 5.7 ^b^	nd	289 ± 20 ^ab^	2.13 ± 0.14 ^c^
Food supplement 2	52.2 ± 3.4 ^bc^	0.475 ± 0.079 ^a^	1.53 ± 0.07 ^b^	285 ± 5 ^d^	139 ± 14 ^a^	0.167 ± 0.014 ^a^	242 ± 16 ^c^	2.70 ± 0.20 ^bc^

*n* = 3; nd—not detected. Values in a column followed by different letters are different at *p* ≤ 0.05 with comparison performed with Tukey’s test. Each value represents the mean of three replicates ± standard deviation.

**Table 2:** Chemical compounds in analyzed samples of *Cordyceps militaris* [mg/100 g d.w.].

Mushroom Material						
Analyzed Compounds	Mycelium from In Vitro Cultures	Fruiting Bodies (Own Cultivation)	Medium after Cultivation	Fruiting Bodies (Commercial Cultivation)	Food Supplement 1	Food Supplement 2
Indole Compounds						
L-Tryptophan	5.84 ± 0.18 ^b^	6.42 ± 0.03 ^b^	nd	8.71 ± 0.03 ^a^	7.75 ± 1.56 ^a^	2.76 ± 0.01 ^c^
5-Hydroxy-L-tryptophan	206 ± 3 ^b^	81.1 ± 4.3 ^e^	70.7 ± 0.7 ^f^	185 ± 3 ^c^	296 ± 3 ^a^	140 ± 4 ^d^
Serotonin	nd	39.4 ± 0.2 ^b^	33.9 ± 1.2 ^c^	nd	nd	122 ± 3 ^a^
Tryptamine	nd	*	nd	nd	nd	nd
Melatonin	*	*	*	*	*	*
Phenolic Compounds						
*p*-Hydroxybenzoic acid	nd	0.038 ± 0.001 ^a^	nd	nd	nd	nd
Quercitrin	2.54 ± 0.01 ^b^	2.10 ± 0.01 ^a^	nd	nd	nd	nd
Rutoside	1.17 ± 0.02 ^a^	0.066 ± 0.002 ^b^	nd	nd	nd	nd
Sterols						
Ergosterol	477 ± 1 ^a^	95.5 ± 0.4 ^e^	259 ± 1 ^b^	142 ± 3 ^d^	155 ± 1 ^c^	4.28 ± 0.01 ^f^
Ergosterol peroxide	*	*	nd	*	*	nd
α-Tocopherol	148 ± 1 ^b^	9.83 ± 0.11 ^d^	nd	96.3 ± 5.2 ^c^	242 ± 1 ^a^	5.87 ± 0.02 ^d^
Other organic Compounds						
Cordycepin	6.34 ± 0.40 ^a^	25.9 ± 0.6 ^d^	24.3 ± 1.2 ^d^	57.5 ± 0.4 ^c^	60.4 ± 0.1 ^b^	81.4 ± 3.1 ^a^
Lovastatin	29.7 ± 1.1 ^c^	30.5 ± 1.5 ^c^	1.90 ± 0.13 ^d^	36.4 ± 0.1 ^b^	53.6 ± 0.3 ^a^	35.4 ± 5.0 ^b^
Ergothioneine	10.4 ± 1.7 ^a^	8.97 ± 0.38 ^ab^	4.21 ± 0.13 ^c^	8.74 ± 0.73 ^ab^	7.93 ± 0.66 ^b^	2.56 ± 0.06 ^c^
L-Phenylalanine	224 ± 2 ^a^	6.56 ± 0.07 ^c^	nd	nd	nd	117 ± 4 ^b^
Antioxidant Activity						
% DPPH	7.85 ± 0.16 ^e^	26.0 ± 0.9 ^b^	18.5 ± 0.5 ^c^	38.4 ± 0.4 ^a^	15.3 ± 0.1 ^d^	17.2 ± 0.5 ^c^
Total phenolic content	34.9 ± 1.2 ^d^	149 ± 2 ^a^	91.3 ± 1.6 ^c^	110 ± 1 ^b^	150 ± 2 ^a^	88.8 ± 1.5 ^c^
After Extraction in Digestive Juices						
Gastric Juice						
L-Tryptophan	nd	17.7 ± 1.1 ^b^	-	41.5 ± 0.1 ^a^	13.7 ± 1.6 ^c^	3.83 ± 0.04 ^d^
5-Hydroxy-L-tryptophan	15.2 ± 1.8 ^c^	10.4 ± 0.8 ^d^	-	21.3 ± 2.0 ^b^	37.3 ± 0.7 ^a^	11.3 ± 0.1 ^d^
Melatonin	nd	*	-	nd	nd	*
Ergosterol	4.63 ± 0.01 ^c^	4.81 ± 0.01 ^b^	-	4.69 ± 0.01 ^c^	7.15 ± 0.07 ^a^	4.49 ± 0.01 ^d^
Ergosterol peroxide	*	*	-	*	*	*
Cordycepin	1.68 ± 0.02 ^a^	nd	-	nd	nd	nd
Lovastatin	nd	*	-	nd	*	nd
Ergothioneine	21.9 ± 1.0 ^b^	28.3 ± 0.8 ^a^	-	19.4 ± 0.3 ^c^	22.5 ± 1.5 ^b^	2.82 ± 0.03 ^d^
L-Phenylalanine	208 ± 1 ^a^	nd	-	nd	nd	26.2 ± 0.3 ^b^
Intestinal Juice						
L-Tryptophan	4.07 ± 0.06 ^c^	7.63 ± 0.03 ^a^	-	7.44 ± 0.08 ^b^	7.42 ± 0.07 ^b^	nd
5-Hydroxy-L-tryptophan	21.8 ± 2.7 ^a^	17.1 ± 0.8 ^b^	-	17.6 ± 1.2 ^b^	22.3 ± 0.5 ^a^	3.40 ± 0.27 ^c^
Melatonin	*	nd	-	*	*	nd
Ergosterol	4.57 ± 0.01 ^c^	4.63 ± 0.02 ^b^	-	4.63 ± 0.01 ^b^	8.14 ± 0.04 ^a^	4.50 ± 0.01 ^d^
Ergosterol peroxide	*	*	-	*	*	*
Cordycepin	8.13 ± 0.24 ^c^	25.8 ± 0.8 ^a^	-	25.9 ± 0.1 ^a^	20.5 ± 0.1 ^b^	7.03 ± 0.01 ^d^
Lovastatin	*	nd	-	*	*	nd
Ergothioneine	2.94 ± 0.01 ^c^	1.82 ± 0.13 ^d^	-	8.53 ± 0.50 ^a^	5.95 ± 0.20 ^b^	1.43 ± 0.01 ^d^
L-Phenylalanine	18.4 ± 0.4 ^a^	nd	-	nd	nd	nd

*n* = 3; *—trace amount; nd—not detected; -—not marked; % DPPH—represents percentage of DPPH reduction; total phenolic content was calculated based on the calibration curve of gallic acid and expressed as gallic acid equivalents (GAE), in mg/100 g d.w. of mushroom material. Values in a row followed by different letters are different at *p* ≤ 0.05 with comparison performed with Tukey’s test. Each value represents the mean of three replicates ± standard deviation.

## Data Availability

Data are contained within the article.
